# “Well, to Be Honest, I Don’t Have an Idea of What It Might Be”—A Qualitative Study on Knowledge and Awareness Regarding Nonmelanoma Skin Cancer

**DOI:** 10.3390/curroncol30020177

**Published:** 2023-02-15

**Authors:** Luisa Leonie Brokmeier, Katharina Diehl, Bianca Annika Spähn, Charlotte Jansen, Tobias Konkel, Wolfgang Uter, Tatiana Görig

**Affiliations:** 1 Department of Medical Informatics, Biometry and Epidemiology, Friedrich-Alexander-Universität Erlangen-Nürnberg (FAU), 91054 Erlangen, Germany; 2 Medical Faculty Mannheim, Heidelberg University, 68167 Mannheim, Germany

**Keywords:** keratinocyte carcinoma, nonmelanoma skin cancer, health competence, risk perception

## Abstract

Nonmelanoma skin cancer (NMSC) is the most common cancer type in Western industrialized countries. However, research into the knowledge and awareness in the general population regarding NMSC is still scarce. This qualitative study aims to fill this research gap. Face-to-face, semi-structured interviews with 20 individuals aged 55–85 years were conducted between February and October 2020. Transcribed interviews were analyzed using qualitative content analysis. The term “white skin cancer”—the German colloquial term of NMSC—was well-known, but the incidence was underestimated. None of the participants could give a precise definition of NMSC, and various alterations in the skin were, partially wrongly, stated as potential signs for NMSC. As risk factors for NMSC, solar radiation, and fair skin type were mentioned most often. The perceived individual risk of developing NMSC and risk compared to individuals of the same age and gender were low in our sample. Own knowledge about NMSC was mostly perceived to be insufficient, and the majority of the sample would like to receive more information on NMSC. Our results emphasize a need to inform about the signs and risks of NMSC not only in the studied older age group but also in younger people.

## 1. Introduction

Nonmelanoma skin cancer (NMSC) is the most common form of cancer in industrialized countries [1,2]. NMSC comprises basal cell carcinoma (BCC) and squamous cell carcinoma (SCC), together with various (very) rare tumors [3,4]. NMSC is diagnosed in over 6.3 million new cases each year worldwide [5]; however, further data sources indicate a possible underestimation of numbers [6], which may be caused by incomplete registration practice [2]. The incidence of NMSC has been constantly rising over recent decades and is expected to increase further [7]. Costs for medical care for NMSC represent a huge burden for health systems. In the US, the average annual treatment costs were estimated at USD 4.8 billion during 2007–2011 [8].

This epidemiological and health economic burden emphasizes the need for the prevention of NMSC. In the UK, treatment costs could be significantly reduced if primary prevention measures were established in the population, as suggested by a modeling study [9]. In health psychology, two major factors were shown to be related to individuals’ preventive behavior: knowledge and awareness of the disease [10,11], and perceived own risk of developing the disease [12,13]. At present, studies investigating the knowledge about NMSC in the general population are scarce.

Some studies have focused on knowledge and awareness regarding skin cancer in general. In a Swedish sample, the incidence of skin cancer and its impact on general health was underestimated, even though solar radiation was known to be a risk factor [14]. In addition, an international survey found widely varying levels of knowledge regarding risk factors. On average, 36% of respondents knew at least five out of six risk factors for skin cancer, ranging from 14% in Russia to 52% in Greece [15]. Only a few studies have explicitly investigated knowledge about NMSC in the general population. In international studies from 2004 to 2005, only 22% of the sample questioned in a telephone interview were familiar with BCC [16,17]. A qualitative study with farmers found little specific knowledge about NMSC, although the majority had heard about other skin cancers than malignant melanoma (MM) before [18]. However, there have been no more detailed and recent findings on knowledge about NMSC in the general population.

Besides knowledge, risk perception can influence protective behavior [19]. A nationwide survey in Germany has explored the perception of the risk of developing skin cancer compared to others of the same age and gender and showed that almost half of the sample (43.3%) believed themselves to be at low risk, which can be considered unrealistically optimistic [20]. In a Swedish study focusing on risk perception of NMSC specifically, 13% perceived themselves at a lower risk of developing NMSC compared with others of the same age [14]. In an international comparison, concern about NMSC ranged widely depending on the nationality of respondents (30% of Germans to 67% of Italians) [16,17]. The conflicting evidence on risk perception regarding NMSC calls for an updated and detailed assessment.

To date, there is no study extensively assessing what the German population knows about NMSC and how they perceive their personal risk. Therefore, the aim of this study was to qualitatively explore: (1) familiarity with the term NMSC (i.e., having ever heard of it) and understanding of NMSC; (2) knowledge about the signs and risk factors of NMSC; and (3) perception of own risk for developing NMSC.

## 2. Materials and Methods

An exploratory qualitative research approach using semi-structured interviews was used to answer research questions. In the following, study procedures, data collection and analysis, eligibility of participants and study team are described, as recommended for reporting qualitative research (COREQ, [21]). 

### 2.1. Study Population

A convenience sample of 20 subjects aged 55 years and older was recruited by the interviewer based on the snowballing technique. Following the principles of theoretical saturation, we stopped recruitment after the 20th interview. Participants had to be fluent in German and able to give informed consent. The recruitment of participants did not explicitly focus on whether individuals had ever been diagnosed with NMSC. No further inclusion or exclusion criteria were applied. An equal distribution by gender was aimed at.

### 2.2. Data Collection

The interview guide for this study was designed in four steps—collecting, probing, sorting, and sub summarizing [22]—and included open-ended questions classified into five thematic blocks: “General information on NMSC”, “Knowledge about NMSC”, “Differences between NMSC and MM”, “Risk factors and potential risk behaviors regarding NMSC”, and “Perception of own knowledge and need for clarification”. In Germany, the term “white skin cancer” (German: “weißer Hautkrebs”) is the more familiar term for NMSC to lay people, and the term “black skin cancer” (German: “schwarzer Hautkrebs”) is used for MM. Therefore, we used these terms in our interview guide. When reporting results in this manuscript, we used the scientific terms “NMSC” and “MM”. Additionally, sociodemographics, sources of information on health issues, and previous skin cancer diagnosis were gathered in a standardized manner during the interview. Fitzpatrick skin type [23] was assessed by the interviewer.

Interviews were conducted by B.A.S. (female paramedic and medical student). She was trained extensively in interviewing and active listening by K.D. (female social scientist and experienced interviewer) before conducting the interviews between February and October 2020. The training interviews served as pilot tests of the interview guide. Prior to the interview, all participants were informed about data protection, the study’s procedure, and its voluntary nature. After the interviews, they received a EUR 15 book store voucher as reimbursement for their time. Interviews were conducted face-to-face, and the audio was recorded at a place suggested by the participants where they felt comfortable talking. The interviews lasted on average 34 min 9 s (SD = 9:43, range 22:16–56:57 min).

This study was approved by the ethics committee II of the Medical Faculty Mannheim, Heidelberg University (ethical approval code: 2019 732N).

### 2.3. Data Analysis

Interviews were transcribed verbatim [24] and then analyzed using structuring qualitative analysis following Mayring [25] using MAXQDA (VERBI GmbH, Berlin, Germany, Version 18.2.0). We used a set of main codes developed a priori based on the interview guide [26]. This set was further complemented during the coding process. All interviews were independently coded by two coders (B.A.S. and C.J.). Disagreements were discussed and resolved by consensus in each case. Findings were summarized descriptively. Descriptive data were analyzed using IBM SPSS Statistics Version 28 (IBM, Armonk, NY, USA).

## 3. Results

Twenty individuals aged 55–85 years (M = 69.9, SD = 11.7) were interviewed; ten interviewees were female (50%). Five (25%) had a migration background. Eleven participants (55%) indicated having high- (German *Abitur*, equivalent to A level/SAT exam) or medium-level (German *Realschule*, equivalent to 5th-year secondary education) school education (Table 1). Twelve participants (60%) were already retired. Most interviewees were living with their spouses (70%).

All participants had skin types I to III. Six participants (30%) reported a prior skin cancer diagnosis, of which four were diagnosed with NMSC, one with NMSC and MM, and one with MM. Nine individuals (45%) knew someone in their social environment who had been diagnosed with NMSC (Table 1).

For most participants (70%), their general practitioner was the first contact person regarding health-related questions, followed by “spouse” (20%). Only one person indicated turning to a medical specialist and one to relatives to get advice on health-related issues. The most frequently named source for health-related information was newspapers (45%), followed by the internet (35%). Two individuals (10% each) indicated television and magazines as their preferred sources for information.

### 3.1. Knowledge about NMSC

#### 3.1.1. Familiarity with the Term NMSC and Definitions

The majority of interviewees (*n* = 16) responded that they had heard of the term “white skin cancer” (the common term for NMSC in Germany) before the interview (Subject (S)07: “*Yes*, *I’ve heard it before. Uhm*, *in connection with uhm visits to my dermatologist*”), while four reported to have never heard about it. However, one of these four participants had heard the term BCC before (S06).

None of the interviewees were able to give a precise definition of NMSC, and four admitted to having no idea at all (S05: “*Well*, *to be honest*, *I don’t have an idea of what it might be*”). While two defined NMSC incorrectly as a benign tumor or an early stage of MM, most interviewees (*n* = 14) gave a vague, yet not incorrect, definition (i.e., “[…] *pathological changes of certain skin areas*, *which I however uhm (.) would not necessarily be able to identify as such myself*”, S07).

#### 3.1.2. Knowledge about Early Signs of NMSC

While two interviewees thought dark spots or alterations in nevi were signs of NMSC, most individuals thought other changes in the skin, e.g., reddish, bleeding, rough, or itching spots (see Supplementary Table S1), would indicate NMSC. Three interviewees believed white or light-colored discolorations of the skin were signs of it accordingly.

#### 3.1.3. Risk Factors for NMSC

Solar radiation was recognized by all participants as a potential risk factor for NMSC (e.g., S06: “*Well*, *possibly also sun/I mean UV light*, *sun exposure and tanning beds*”) and tanning bed use by seven interviewees (e.g., S07: “*So*, *um tanning beds are*, *I think*, *well*, *are a source of danger*”). While seven individuals saw light skin type as an increased risk for NMSC (e.g., S14: “*So as far as I know, um, are, are light skin types more damaged by sun exposure*”), two dismissed it as a risk factor (S17: “*Um*, *no*, *I think*, *skin is skin and it will behave similarly*”). Contact with chemicals was mentioned as an NMSC risk factor (*n* = 6, i.e., S14: “*I could also imagine, perhaps if someone works with chemicals in their job or something, their whole life, that that might also be conducive [to NMSC]*”), as well as genetic factors other than skin type (*n* = 6, i.e., S08: “*(..) Well I could still imagine that it’s also a little bit in heredity*, *so that/ heredity is a part*, *because that’s also more common in my family.*”). Furthermore, health-related behaviors, such as smoking, unhealthy diet, alcohol consumption, “*various medications*” (S10), and “*vulnerability of the immune system*” (S16), were sporadically mentioned as risk factors.

#### 3.1.4. Perception of Own Knowledge about and Desire for More Information on NMSC

Most participants (*n* = 15) perceived their knowledge about NMSC was insufficient (e.g., S12 stated: “*No*, *I don’t know much about it*”). Only two interviewees perceived themselves as well-informed regarding NMSC (e.g., S14: “*[…] Let me state it like this*, *I believe that I’m fairly good informed*”), and three rated their knowledge level as medium (e.g., S09: “*Actually I think I am at least / I think that I know enough about it*”). Consequently, almost the entire sample (*n* = 17) indicated they would like to receive more information on NMSC. In particular, respondents would appreciate more information on the appearance of NMSC, its consequences and severity, development and prevention, and its treatment options.

### 3.2. Awareness Regarding NMSC

#### 3.2.1. Estimated Prevalence of NMSC

When asked to estimate the prevalence of NMSC in comparison to MM, nine participants thought it is less prevalent than MM (S06: “*I’m not informed, but I would say the [MM] is more frequent*”), while seven correctly assumed the opposite (S14: “*I believe that [NMSC] is more frequent*”). Four participants had difficulties answering this question and could not give an estimation.

#### 3.2.2. Perceived Dangerousness of NMSC

About half of the sample (*n* = 11) perceived NMSC as threatening (S08: “*[…] And then I realized somehow*, *it was a threat to me after all*”). However, seven interviewees did not take this disease seriously (I: “*And how threatening do you perceive [NMSC] to be?*”, S13: “*Oh*, *at my age*, *not at all*, *actually*”). One person was indecisive.

#### 3.2.3. Perception of Risk of NMSC

Risk perception regarding one’s own health.

**Figure 1:**
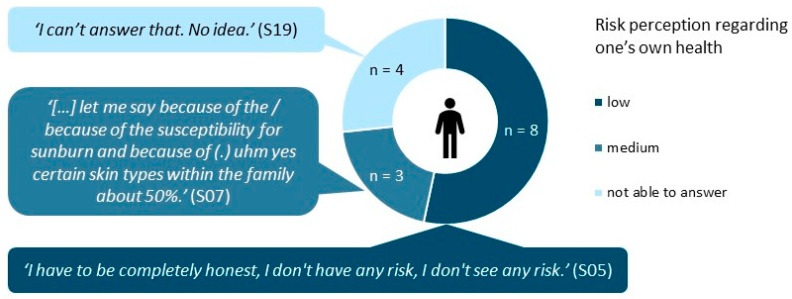
Participants’ answers to “How would you estimate your risk to develop NMSC?”. Numbers based on participants without a self-reported diagnosis of NMSC (*n* = 15). NMSC: nonmelanoma skin cancer.

We asked interviewees who had not been diagnosed with NMSC according to their self-reports (*n* = 15) to estimate their own risk for developing NMSC. About half indicated a low individual risk, while the other participants estimated their risk as medium or were unable to estimate their risk (see Figure 1). Those rating their risk as medium had all indicated they had used tanning beds in the past.

Risk perception compared with persons of the same age and sex.

**Figure 2:**
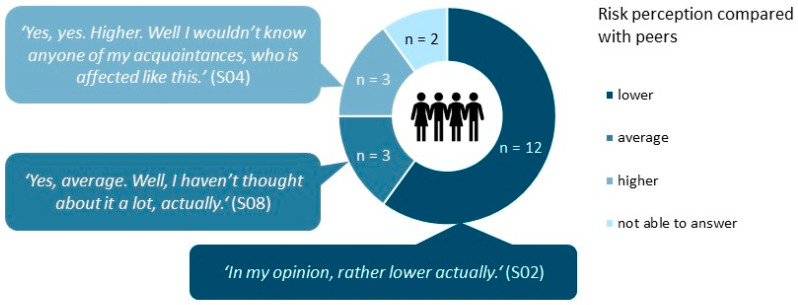
Participants’ answers to “Compared with an individual of same sex and age, how would you estimate your risk to develop NMSC?”. *n* = 20. NMSC: nonmelanoma skin cancer.

When asked to estimate their own risk of developing NMSC compared to a person of the same age and sex, even more interviewees perceived their risk as rather low (see Figure 2). All three respondents who estimated their risk as higher than that of peers already had a diagnosis of NMSC.

## 4. Discussion

To our knowledge, this study is the first to qualitatively and broadly explore the knowledge about and risk perception regarding NMSC in the general population. While the majority indicated having heard of NMSC before, knowledge regarding the definition, signs, dangerousness, and prevalence of NMSC was rather low. Even though participants knew about the most important risk factors for NMSC, they perceived their own risk of developing NMSC, especially compared to others, as rather low. These findings, combined with the wish for more information on NMSC in our sample, call for more information and education on NMSC.

### 4.1. Knowledge about NMSC

Most respondents had heard the term “white skin cancer”—a German colloquial term for NMSC—before, whereas correct definitions could not be given, which is in line with previous research [18]. However, it is not surprising that definitions and signs of NMSC could not be given clearly and correctly by questioned lay persons, since the signs of NMSC can vary widely [27]. The most common form of NMSC is BCC, typically manifesting as a pink, shiny to pearly papule [28], and this sign was not mentioned by anyone in our sample. Furthermore, some of the participants assumed white or light skin alterations to be signs of NMSC, probably owing to the German common term for NMSC (i.e., “white skin cancer”). Some respondents mentioned dark discolorations or alterations in nevi as signs for NMSC. This might indicate that they may have thought of MM and might not even be aware of the different forms of skin cancer. Thus, more general information on typical signs of NMSC is necessary to enable the general population to identify potential NMSC timely and to consult specialists for further examination. In view of the misleading denomination of NMSC in German as “white skin cancer”, it should be pointed out in these materials that this term is a misnomer for NMSC—although hypopigmentation may rarely be present in BCC [29].

Contradicting the findings from the qualitative study by Zink et al. [18], which was conducted amongst farmers, our participants were all aware of the most important environmental risk factor for NMSC, i.e., solar radiation. However, participants in the former study were well aware of the protective effects of sunscreen against skin cancer [18]. This might indicate that they implicitly knew about the risk for NMSC through solar radiation. Additionally, patients with actinic keratosis (AK) were aware of the association between sunlight and AK as a precursor of SCC [30,31]. Furthermore, high awareness of the relationship between solar radiation and all skin cancers has been found in other studies [32,33,34]. In contrast, awareness of other risk factors, such as genetics, was rather low in our sample, as well as in a Brazilian one [33]. Even the phenotypic skin type—clearly dependent on genetic factors—was mentioned only by a few participants as a risk factor for NMSC, although skin types I and II according to Fitzpatrick’s classification were shown to be risk factors for both BCC and SCC [35,36]. However, unlike the other genetic predispositions, patients can recognize the skin type themselves, and use it to adequately protect themselves. Thus, information and education materials should also focus on easily identifiable and modifiable risk factors for NMSC.

### 4.2. Risk Perception of NMSC

Most respondents perceived their risk of developing NMSC as low, especially compared with the risks of peers. This so-called optimistic bias has been found in previous research as well [14,20]. Furthermore, our sample consists of older individuals, who seem to be optimistically biased. This is in line with previous research. In an American study, older survey participants perceived their risk for NMSC in comparison to peers as being lower than younger respondents [37]. A similar trend was found in a German representative survey: the older age of participants was associated with lower risk perception for skin cancer [34]. This seems worrying because the risk for NMSC increases with age [38]. Risk perception could be an important factor for behavior change, as outlined in psychological behavior prevention models (e.g., the Health Action Process Approach and the Transtheoretical Model of Behavior Change [11]). An awareness of the high risk of developing NMSC might, therefore, lead to more adherence to sun-protective behavior. Because risk perception regarding skin cancer seems to be rather low, it should be targeted in future prevention and information campaigns. As young people can lower their individual risk for NMSC by modifying their behavior towards, for instance, better UV protection [39], they should also be the focus of future campaigns. Moreover, future studies should also focus on a younger population to explore potential biases in risk perception for NMSC.

When asked about their risk perception regarding their own health, four respondents were unable to give an estimation. However, when asked to compare their own risk for NMSC with the risks of their peers, this proportion was lower. This might be due to the fact that the former question was asked at the beginning of the interview, while the latter was asked at the end, after having talked about various aspects of NMSC. According to the availability heuristic, decisions are influenced by the information that is most salient in the mind [40]. The same was shown to be relevant for forming risk perceptions [41]. Therefore, after having recalled the knowledge about NMSC, participants might have felt more competent in estimating their own risk (i.e., in comparison to peers). This suggests that reminding lay persons of facts about NMSC may trigger them to reevaluate their risk perception.

### 4.3. Desire for More Information on NMSC

Most participants were aware of their lack of knowledge regarding NMSC and accordingly expressed a desire for more information. As became evident in the interviews, this information should include an easily comprehensible definition of and possible signs for NMSC, as well as the actual prevalence of NMSC and the probability of falling ill with it. Moreover, information on risk should be conveyed sensibly because there seems to be an unrealistically optimistic risk perception regarding NMSC in the population, as found before regarding skin cancer in general [20]. Channels for information distribution could be general or dermatological practices, newspapers, and the internet since these media were considered the most important with regard to health-related issues. Emphasis should be on the first because the majority of our sample named general practitioners as their go-to source for information regarding health.

### 4.4. Limitations

Some limitations in our study should be considered when interpreting the results. Firstly, due to the qualitative origin of this study, we interviewed a sample of 20 subjects, and our findings are, therefore, not generalizable. However, this qualitative exploratory design was necessary to gain a first insight into what is known by lay persons. The lack of knowledge this revealed should be validated on a large scale and supported with quantitative data. Answers given by our sample can be used to create items for such a large-scale survey. Secondly, our sample comprised older citizens due to the high prevalence in this age group, and it may be rather homogeneous due to the use of the snowballing technique for recruitment. The snowballing sampling technique might, thus, lead to selection bias [42,43]. However, it is a common sampling method in qualitative studies [43] and is frequently used (e.g., [44,45]). Despite the potential shortcomings of this method, we have reached a sample that is quite balanced in terms of sex, migration background, and education. Although we focused on older people, the prevention of NMSC would be most effective if started at a young age, which is why the knowledge and risk perception of the younger population should be addressed in future research [46]. Thirdly, due to our sample size, we were not able to associate the identified unrealistically optimistic risk bias regarding NMSC with any personal characteristics of the participants. For instance, it would be interesting for future studies to link skin type with personal risk perception to analyze the adequacy of individual perception. Therefore, this aspect requires verification in a larger sample, and the findings could be stratified by sociodemographic characteristics. Finally, a bias due to the self-selection of participants or social desirability within the participants’ reports cannot be ruled out.

## 5. Conclusions

This qualitative study revealed considerable knowledge gaps regarding NMSC in a German sample comprising lay people of 55 years and older, as well as a low risk perception. Prevention and information measures should be planned with these results in mind. For example, skin cancer screening examinations, which are part of the regular healthcare services offered in Germany, could include more detailed information on NMSC. Because NMSC frequently occurs in the head and neck area, hairdressers could also be motivated and trained to detect early signs of skin cancer and to communicate them to their clients [47]. Both preventative measures and information distribution to raise awareness for NMSC will be needed.

## Figures and Tables

**Table 1:** Characteristics of participants of the qualitative interviews (*n* = 20).

ID	Age	Sex	Migration Background	Highest Education ^1^	Retirement	Living Situation	Fitzpatrick Skin Type	Diagnosis of Skin Cancer ^2^	NMSC in Social Environment
S01	73	Female	No	Medium	Yes	Alone	III	No	No
S02	82	Male	No	Low	Yes	Alone	II	No	No
S03	83	Female	Yes	Low	Yes	With children	III	NMSC	Yes
S04	80	Female	No	Low	Yes	With spouse	II	NMSC	Yes
S05	83	Female	No	Low	Yes	With spouse	III	No	No
S06	85	Male	No	High	Yes	With spouse	III	No	No
S07	54	Male	No	High	No	With spouse	III	No	Yes
S08	57	Female	No	High	Yes	With spouse	I	NMSC	No
S09	56	Male	No	High	No	With spouse	II	MM	Yes
S10	58	Female	Yes	High	No	With spouse	III	No	Yes
S11	77	Male	Yes	Low	Yes	With spouse	II	AK, NMSC, MM	Yes
S12	85	Female	No	Low	Yes	With spouse	III	No	No
S13	83	Male	Yes	High	Yes	With spouse	II	NMSC	No
S14	63	Male	No	High	No	Alone	III	AK	No
S15	55	Male	No	Medium	No	Alone	II	No	Yes
S16	64	Male	Yes	Other	No	Alone	III	No	No
S17	75	Male	No	High	Yes	With spouse	III	No	No
S18	59	Female	No	Low	No	With spouse	III	No	Yes
S19	61	Female	No	Medium	No	With spouse	II	No	Yes
S20	65	Female	No	Low	Yes	With spouse	III	No	No

^1^ High: German “Abitur/Fachabitur”, equivalent to A level/SAT exam; medium: German “Realschulabschluss”, equivalent to 5th year secondary; and low: German “Hauptschulabschluss”, equivalent to basic secondary schooling. ^2^ Diagnosis of skin cancer according to self-reports of participants. AK: actinic keratosis; MM: malignant melanoma; and NMSC: nonmelanoma skin cancer.

## Data Availability

Data are available from the corresponding author upon reasonable request.

## Supplementary Materials

The following supporting information can be downloaded at: https://www.mdpi.com/article/10.3390/curroncol30020177/s1, Table S1: Signs of NMSC reported by interviewees.
