# Supplementary material for: “Well, to Be Honest, I Don’t Have an Idea of What It Might Be”—A Qualitative Study on Knowledge and Awareness Regarding Nonmelanoma Skin Cancer
Source: Curr Oncol. 2023 Feb 15;30(2):2290–9. doi: 10.3390/curroncol30020177 (PMC9955472; doi:10.3390/curroncol30020177)
Supplement: Supplementary file 1 [file curroncol-30-00177-s001.zip › curroncol-2146242-supplementary.pdf]

Current Oncology

MDPI

Supplementals

"Well, To Be Honest, I Don't Have an Idea of What It Might Be"—A Qualitative Study on Knowledge and Awareness Regarding Nonmelanoma Skin Cancer

Luisa Leonie Brokmeier [1], Katharina Diehl [1,*], Bianca Annika Spähn [2], Charlotte Jansen [2], Tobias Konkel [1], Wolfgang Uter [3] and Tatiana Görig [1]

Table S1. Early signs of NMSC reported by interviewees.

Type of signs	Quotes stated by participants
Changes of skin not further defined	S01: '*have no idea, I can't, umm, estimate that. Let me say […] if I notice changes on the skin […], I will go to the dermatologist and get it checked.*' <br>S10: '*To be honest, I don't have a lot of knowledge, but I think like how my mother had it, that was something like a uhm / well something similar to a swelling on the skin […]*' <br>S17: '*[…] they would be […] of a different color and of a different texture, how the skin feels, from normal skin to a cancer area, has to be different.*' <br>S18: '*The normal skin as well. It could be that on a normal area there is a sudden change / so not just these birthmarks.*'
Itching, rough skin	S04: '*Umm, rough skin. And I don't know, up there I had, but that was some kind of bump, that was correct //*' <br>S07: '*Umm well, I think, changes of the skin, possibly also itching or so.*' <br>S11: '*You can see it somehow. Something appears or maybe it itches or so and then you can see that there is something. Or there is a redness, and so on.*' <br>S12: '*Um, I think one will maybe realize that there is perhaps some rough skin spots or something like that or maybe it also itches, I don't know that exactly.*' <br>I: '*[…] so, indicators, would you know any […]?*' S02: '*Oh god no, well I can only imagine that maybe there is some kind of itching, that one has to scrub and scratch constantly. […] I can't imagine how that is.*' <br>S16: '*Ah yes, on the one hand the, the skin changes. Skin changes and (.) I guess there is also some kind of um, itching or something like that as well/*'
Reddish skin areas / skin areas that	S06: '*The symptoms, that there are, well, areas on the skin, which won't heal or which, um, look wound-like, and (.) abnormal*'

bleed or heal poorly	S03: *'Yes. Like red pimples or (.) whatever that was and it got bigger. And after I had showered or rubbed it with the towel, it also started bleeding.'*<br><br>S08: *'Umm, well, first I thought I had a wart. But then it got infected and, um, it was painful and turned quite red, so I went to the dermatologist. […] I don't know, whether it always looks like this, I can't say. But if it looked like it did on me or if I felt it, I would know that it's that.'*<br><br>S11: *'You can see it somehow. Something appears or maybe it itches or so and then you can see that there is something. Or there is a redness, and so on.'*<br><br>S13: *'(..) (sighs) Not really, I just have, I can only speak for myself, I have realized it because of this small pimple and also up here, there was some sort of small growth, but other than that I didn't notice anything, no.'*
Nevi; dark discolorations of skin areas	S05: *'Yes, probably some sort of change on the skin, (..) well here I have those spots for example, these black ones, yes, with these my doctor always tells me / big, noticeable ones, but the doctor always tells me that those are not a big deal. It's only a little blemish.'*<br><br>S18: *'Yes but those / but not just those brown spots, I / I think, […] if one has birthmarks that are changing, which then […] look completely different.'*
Light discolorations of skin areas	S09: *'Well, um, skin spots becoming lighter.'*<br><br>S19: *'Like I said, I think there are perhaps white spots, which don't look exactly round or which have a / which have a different structure compared to pigmentation disorders or spots that are usually on the skin.'*<br><br>S20: *'Yes, like I said, light skin spots, on the arms or the stomach, the legs, the buttocks, where it can appear, I would say light spots on the skin.'*
